# Association of Diabetes Related Complications with Heart Rate Variability among a Diabetic Population in the UAE

**DOI:** 10.1371/journal.pone.0168584

**Published:** 2017-01-20

**Authors:** Ahsan H. Khandoker, Haitham M. Al-Angari, Kinda Khalaf, Sungmun Lee, Wael Almahmeed, Habiba S. Al Safar, Herbert F. Jelinek

**Affiliations:** 1 Department of Biomedical Engineering, Khalifa University, Abu Dhabi, United Arab Emirates; 2 Khalifa University Center of Biotechnology, Abu Dhabi, United Arab Emirates; 3 Institute of Cardiac Science, Sheikh Khalifa Medical City, Abu Dhabi, United Arab Emirates; 4 Heart and Vascular Institute, Cleveland Clinic, Abu Dhabi, United Arab Emirates; 5 School of Community Health, Charles Sturt University, Albury, New South Wales, Australia; 6 Australian School of Advanced Medicine, Macquarie University, Sydney, New South Wales, Australia; Temple University, UNITED STATES

## Abstract

Microvascular, macrovascular and neurological complications are the key causes of morbidity and mortality among type II diabetes mellitus (T2DM) patients. The aim of this study was to investigate the alterations of cardiac autonomic function of diabetic patients in relation to three types of diabetes-related complications. ECG recordings were collected and analyzed from 169 T2DM patients in supine position who were diagnosed with nephropathy (n = 55), peripheral neuropathy (n = 64) and retinopathy (n = 106) at two hospitals in the UAE. Comparison between combinations of patients with complications and a control diabetic group (CONT) with no complication (n = 34) was performed using time, frequency and multi-lag entropy measures of heart rate variability (HRV). The results show that these measures decreased significantly (p<0.05) depending on the presence and type of diabetic complications. Entropy, (median, 1^st^- 3^rd^ interquartile range) for the group combining all complications (1.74,1.37–2.09) was significantly lower than the corresponding values for the CONT group (1.77, 1.39–2.24) with lag-1 for sequential beat-to-beat changes. Odds ratios (OR) from the entropy analysis further demonstrated a significantly higher association with the combination of retinopathy and peripheral neuropathy versus CONT (OR: 1.42 at lag 8) and an even OR for the combination of retinopathy and nephropathy (OR: 2.46 at lag 8) compared to the other groups with complications. Also, the OR of low frequency power to high frequency power ratio (LF/HF) showed a higher association with these diabetic-related complications compared to CONT, especially for the patient group combining all complications (OR: 4.92). This study confirms that the type of microvascular or peripheral neuropathy complication present in T2DM patients have different effects on heart rate entropy, implying disorders of multi-organ connectivity are directly associated with autonomic nervous system dysfunction. Clinical practice may benefit from including multi-lag entropy for cardiac rhythm analysis in conjunction with traditional screening methods in patients with diabetic complications to ensure better preventive and treatment outcomes in the Emirati Arab population.

## Introduction

Type II diabetes mellitus (T2DM) is a multifactorial disease characterized by altered glucose metabolism that can affect organ function either directly or indirectly through oxidative stress and inflammatory mechanisms linked to hyperglycemia [1]. Oxidative stress and inflammation are considered as the main causative factors of diabetes complications including renal, retinal vascular and neurological impairments [2, 3]. However, the mechanisms for the development of diabetic related complications are most likely multifactorial and involve in addition to oxidative stress and inflammation, environmental and lifestyle factors, as well as genetic predisposition [4].

T2DM complications do not always occur in isolation but are often found as a group in patients, especially in those who do not have good glucose control, and most often involve autonomic dysfunction. All major organs of the body are modulated by the autonomic nervous system (ANS) and ANS dysfunction is a major cause of increased morbidity and mortality in T2DM. Approximately half of the nephropathy patients attending secondary and tertiary care experience autonomic impairment and cardiopathy [5]. Similarly, diabetic retinopathy has been associated with cardiac autonomic dysfunction in both type 1 [6] and type 2 diabetes mellitus patients [7]. Diabetic peripheral neuropathy (DPN) and peripheral vascular disease have also been associated with symptoms of distal sympathetic autonomic neuropathy [8].

The sympathetic and parasympathetic parts of the ANS are both affected by increased blood glucose levels, oxidative stress, and inflammation processes, potentially leading to multiple organ dysfunction and cardiac autonomic neuropathy (CAN) [9]. CAN is characterized by an altered cardiac rhythm due to the initial changes in the parasympathetic followed by sympathetic modulation of the cardiac rhythm [10–12]. Meta-analyses of published data have demonstrated that reduced cardiovascular autonomic function as measured by heart rate variability (HRV) is strongly (i.e., relative risk is doubled) associated with an increased risk of silent myocardial ischemia and mortality in diabetes patients [13]. However, up to date there are no reports on how single or multiple diabetic complications (DPN, nephropathy and retinopathy) affect cardiac autonomic dysfunction.

HRV analysis is a noninvasive method to assess variations in autonomic nervous system modulation of the heart and has been used previously to evaluate impairments in the sympathetic and vagal tone associated with diabetes, cardiovascular disease, depression, schizophrenia and Parkinson’s disease [14–18].

Our hypothesis is that multi-lag HRV parameters are associated with different combinations of T2DM related complications. Multi-lag heart rate analysis has the advantage of identifying possible contributions of the parasympathetic and sympathetic components of the ANS and therefore may be more sensitive to changes in the ANS associated with different complications. For example, the parasympathetic nervous system influences the heart rate on a beat-by-beat basis up to 10 beats in sequence. Sympathetically driven changes in the heart rate are slower and occur most likely at longer time lags. The aim of this study was twofold: 1) to investigate whether the various diabetic complications and combinations affect HRV and entropy at multiple lags (1–8); and 2) to study if the observed HRV results differ depending on the type of diabetic complication and the combinations of these complications present in the current group of patients. Our findings have confirmed that the types of microvascular complications present in T2DM patients lead to differences in heart rate variability and multi-lag entropy results at lower to higher lags, which suggests that this method may be a useful adjunct to clinical decision making for treatment options related to diabetes complications.

## Materials and Methods

A total of 169 (119 females and 50 males), unrelated diabetic patients who were enrolled during a routine visit to the endocrinology and cardiology clinics at Shaikh Khalifa Medical Center (SKMC) and Mafraq Hospital in Abu Dhabi, United Arab Emirates participated in the study during the period between July 2014 and May 2015. Each volunteer agreed to take part in this study after a briefing session and upon, signing an informed consent form that had been approved by the Institutional Ethics Committee of both hospitals (REC-04062014 and R292 respectively). Inclusion criteria were UAE-born national, diagnosed diabetic patient at one of the hospitals with and without complications, able to give consent, does not have coronary artery disease and above 18 years of age. Exclusion criteria were, current pregnancy, and other pathophysiology such as cancer.

The patients included in the study were all diagnosed with T2DM either from the medical records or reported medication used at the time of the hospital visit. The presence of diabetic associated complications was confirmed by a qualified physician, based on the criteria outlined by the World Health Organization (WHO) consultation group report [19].

The patients in this study were categorized into 8 groups: 1) ALL-C group which included patients with retinopathy, nephropathy and DPN, 2) RNp: included patients with retinopathy and nephropathy, 3) NNp: patients with DPN and nephropathy, 4) RN: patients with retinopathy and DPN, DPNn: patients with DPN, 6) R: patient with retinopathy, 7) Np: patients with nephropathy, and 8) a control group (CONT), which included patients with none of these three complications. A patient was diagnosed with DPN if they presented with (1) foot ulcers, (2) loss of sensation/numbness/burning/tingling in the feet, (3) loss of toe, foot or leg due to diabetes, (4) pain in calf muscles while walking, or (5) peripheral vascular disease in the legs [20]. The presence of nephropathy was determined by urine albumin level higher than 20 μg/min for microalbuminuria and higher than 200 μg/min for macroalbuminuria or if the estimated glomerular filtration rate (eGFR) was less than 60 ml/min/1.73m^2^. Retinopathy was defined as either white or red lesions or both present in the retina according to WHO criteria [21, 22].

### 2.1 Data Collection

Demographic data collection and clinical assessment for each participant were completed at the clinics. ECG signals were recorded for 10 minutes under supine rest and edited using the MLS310 HRV module (version 1.0, ADInstruments, Australia) included in the Chart software package. High frequency noise was removed with a 45Hz low-pass filter and a 0.5 Hz high pass filter adjusted for wandering baseline. Ectopic beats were selected visually and deleted manually. Linear interpolation was used to replace ectopic beats that occur immediately before and after the ectopic interval. Intervals between successive R waves of the QRS complex (i.e., RR intervals in seconds) were calculated using the algorithm developed by Pan and Tompkins [23]. The HRV analysis described in the following sections was performed on 500 RR intervals selected from the middle part of the 10 minute recording [24].

### 2.2 Time and Frequency Domain Analysis

The mean RR (mRR), the standard deviation of normal RR intervals (SDNN), the standard descriptors of the Poincaré plot (SD1 and SD2) [25] were computed for time domain analysis. Spectral analysis was performed using the fast Fourier transform on linearly resampled (1 Hz) time series using Welch’s method with a 50% overlap between adjacent segments [26]. A Hanning window was applied to avoid spectral leakage. Subsequently, spectral powers were binned in the low frequency (LF) band (0.04–0.15 Hz), the high frequency (HF) band (0.15–0.40 Hz). From these bands HF, LF and the LF/HF were determined [27].

### 2.3 Multi-lag Entropy Analysis

In entropy analysis, the RR interval lengths associated with the continuous heartbeat are recorded as a percentile change. The entropy can then be determined from the probability distribution of the percentile change of the RR intervals using information theory such as the information content suggested by Shannon [28].

In conventional entropy analysis, the percentile change of the successive RR intervals with respect to the first RR interval is expressed as the percentage index (PI) defined as:

$$PI\left(i\right)=\;\frac{RR_{i}-RR_{i+1}}{RR_{i}}*100$$ (1)

Entropy is defined from the probability distribution of PI using Shannon’s formula:

$$Entropy=\;\sum_{n}p\left(i\right){\text{log}}_{2}\left(p\left(i\right)\right)$$ (2)

where p(i) is a probability of PI(n) having values in the range i ≤ PI(n)≤ i+1, where i is an integer. Multi-lag entropy differs as it analyses the probability changes of the RR intervals within a set of predefined heart beats, which in the current analysis is set between 1 and 10 beats. For multi-lag entropy analysis, a lag of m points was introduced in Eq (1). Hence, in the multi-lag entropy analysis, PI is expressed as the percentile change of the i-th and i+m-th RR intervals with respect to the i-th RR interval and is defined as:

$$PI\left(i\right)=\;\frac{RR_{i}-RR_{i+m}}{RR_{i}}*100$$ (3)

Where m is an integer and m = 1 represents the conventional entropy analysis. More details on the conventional entropy method can be found in previous work [29, 30].

**Fig 1:**
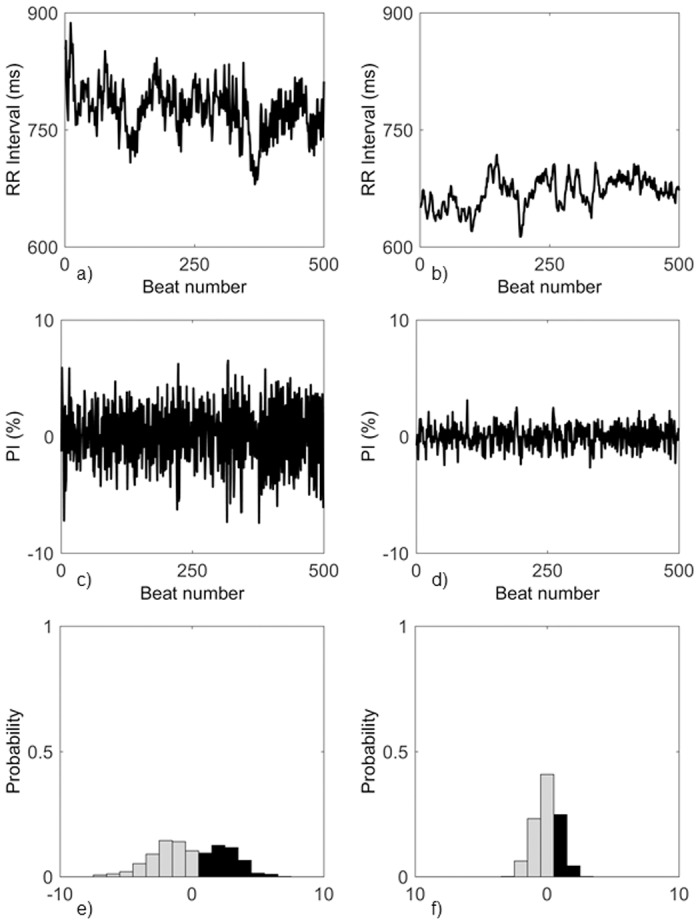
Deriving probability and histogram from RR intervals. RR interval data from a CONT patient (a) and a DPNn patient (b), their PI (c,d) and PI histogram (e,f).

An example of the derived probability of RR intervals of certain lengths occurring in the heart rate tachogram and the derived histogram (for the multi-lag entropy computation) from the CONT and DPNn group is shown in Fig 1.

### 2.4 Statistical Analysis

The Lilliefors test of normality was performed on all features to test for the normal distribution of data. A Kruskal-Wallis nonparametric test of variance was applied for comparing the CONT group and each of the other seven groups mentioned above. We used a parametric analysis of covariance (ANCOVA) to correct for the age and years of diabetes and test feature significance between the CONT group and each of the other groups with complications. A nonparametric ANCOVA (Quade test) was used when the assumptions for parametric ANCOVA were violated [31, 32] Spearman’s Rank-Order Correlation was performed between the HRV features and the demographic data. A binary logistic regression model was used to check association with the HRV features for each of the complication groups compared to CONT and odds ratio (OR) for each of the HRV features was determined. MatLab (R2014b) statistical toolbox was used to perform the statistical analysis.

## Results

The data set used in this study included 135 T2DM patients who were diagnosed with retinopathy, DPN, nephropathy, or combinations of these three complications as well as 34 T2DM patients with no complications. Table 1 summarizes the clinical variables of the patient groups in this study.

**Table 1:** Demographic data for the 8 T2DM groups.

Clinical Variables	CONT (34)	ALL-C (19)	RNp (21)	NNp (4)	RN (27)	DPNn (14)	R (39)	Np (11)
Age (yrs)	55.5 (44,60)	65 (53,74)†§	64 (54,67)†§	60.5 (55.5,62.5)	58 (54.5,66.5)*	54.5 (40,60)	60 (53.5,64)*	66 (54,75)*
DM years	7.5 (4,11)	20 (10,25)†§	16 (5,24.5)†	21 (17.5,26)†§	14 (8,20)†	12.5 (9,20)*	11 (7,16)*	5 (4,18)
BMI (kg/m^2^)	31 (28.9,38.9)	30 (26.8,36)	33.4 (27.6,38.8)	32.1 (30.3,33)	31.8 (27.7,36.8)	30.8 (27.8,36.4)	33.7 (28.4,38)	30.7 (28.3,34.1)
SBP (mmHg)	130 (114,138)	135 (125,142)	135 (127,149)	130 (125,131)	131 (117,136)	125 (112,131)	128 (122,138)	138 (119,153)
DBP (mmHg)	79 (69.5,88.5)	79 (68.5,89)	80 (75,86.5)	84 (70,87)	80 (65,86)	75.5 (71,85)	79 (70,86.5)	77 (69,87.5)
HbA1c (%)	6.8 (6.1,8.9)	7.6 (6,8.7)	7.8 (6.55,8.65)	6.8 (6.55,7.65)	7.2 (6.4,8)	8 (6.95,10.4)	7.55 (6.9,8.8)	7.5 (6.65,8.25)
TC(mmol/L)	4.59 (3.72,5.6)	3.64 (3.1,4.53)*	3.58 (3.12,4.36)*	3.73 (2.81,5.21)	3.54 (2.99,4.3)*	3.55 (3.17,4.49)	3.78 (3.36,4.24)*	3.76 (3.09,4.42)
HDL (mmol/L)	1.27 (1.09,1.63)	1.1 (0.92,1.37)	1.08 (0.95,1.26)*	1.46 (1.05,1.63)	1.16 (0.95,1.57)	1.17 (0.965,1.29)	1.35 (1.08,1.54)	1.45 (1.07,1.59)
LDL (mmol/L)	2.72 (1.73,3.44)	1.75 (1.16,2.6)	1.69 (1.19,2.55)*	1.94 (1.15,3.15)	1.76 (1.21,2.67)*	1.82 (1.58,2.35)	1.88 (1.53,2.17)	1.79 (1.48,1.98)
BGL (mmol/L)	6.65 (5.65,9.35)	7.85 (7.23,9.6)	7.15 (5.9,10.25)	8 (7.35,12.5)	8.5 (6.2,13)	7.8 (6.75,8.4)	8.85 (7.23,11.03)	8.65 (7,10.6)
**Medication**								
Combination drugs	5	7	7	1	11	6	17	3
Incretin mimetics	3	1	4	0	6	3	9	1
Insulin analogs	9	9	11	2	13	9	13	5
Antianxiety, Antidepressive & Antipsychotic	4	0	2	0	3	2	0	0

Values are presented in median (1^st^ Q, 3^rd^ Q). Numbers in brackets represent number of patients in each group. Clinical Variable abbreviations: 1) DM years: years of Diabetes Mellitus, 2) BMI: Body Mass Index, 3) SBP: Systolic Blood Pressure, 5) DBP: Diastolic Blood Pressure, 6) HbA1c: Glycated Hemoglobin Level, 7) TC: Total Cholesterol, 8) HDL: High Density Lipoprotein, 9) LDL: Low Density Lipoprotein, BGL: Blood Glucose Level. Patient group abbreviations: 1) CONT: control group, 2) ALL-C: patients with retinopathy, DPN, and nephropathy. 3) RNp: patients with retinopathy and nephropathy, 4) NNp: patients with DPN and nephropathy, 5) RN: patients with retinopathy and DPN, 6) DPNn: patients with DPN, 7) R: patients with retinopathy, 8) Np: patients with nephropathy.

^†^: significantly different from CONT (p< 0.01)

*: significantly different from CONT (p<0.05)

^§^: significantly different from CONT for the multicomparison test (p<0.05).

Significant differences in the demographic variables (using Kruskal-Wallis test) were observed for age, diabetes mellitus years (DM years), total cholesterol level, high and low density lipoprotein between the groups with different diabetic complications. Age was significantly lower in the CONT group compared to the remaining groups except for N and NNp, which also had a lower mean age but were not significant. DM years was significantly lower in the CONT group as compared to all groups-except for the Np group. Cholesterol levels differed significantly between the control and the diabetic complications and combinations (Table 1). Medication information is categorized into 4 groups: a) Combination drug use (Biguanides, dipeptidyl peptidase-4 inhibitors, sulfonureas and sodium glucose transport inhibitors), b) Incretin mimetics, c) Insulin analogs, d) Antianxiety, antidepressive and antipsychotic medications (Table 1). The values in the table indicate the number of patients who were using that type of medication in each group.

Without correcting for confounding effects, only the RN group entropy results with lags 5–8 were significantly lower than CONT (p<0.05). No other comparisons were significant. To correct for any possible confounding effects we applied ANCOVA and included age and DM years as covariates in the model and show the results in Table 2. Comparisons between the HRV time, frequency and entropy features for the CONT group and multiple combinations of diabetes complications are shown in Table 2. SD1 was significantly lower in the groups that included nephropathy patients as compared to CONT. In contrast, SD2 was significantly lower for the groups that presented with retinopathy and combined with either nephropathy or DPN. DPN as a single complication was not significantly different from the CONT group for any HRV features. However in combination with retinopathy or nephropathy, significantly lower HRV results were observed.

**Table 2:** Results for 8 T2DM groups using time, frequency and multi-lag entropy features.

Feature	CONT	ALL-C	RNp	NNp	RN	DPNn	R	Np
mRR (ms)	746 (650,819)	776 (702,933)	801 (680,859)	880 (757,996)	720 (687,809)	692 (639,772)	737 (694,820)	716 (700,749)
SDNN (ms)	22 (14,27)	19 (14.5,27.4)	18 (13,25.7)*	19 (10.4,28.7)	17 (11.5,22.6)†	21 (18,35)	17 (14,28)	13 (12,36)
SD1 (ms)	8.2 (5.5,12)	7.8 (5.0,11)*	6.8 (5.2,12)	5.7 (3.0,12)*	6.6 (4.1,9.52)*	7.5 (6.5,14)	8.1 (6.2,11)	5.4 (3.7,9.1)*
SD2 (ms)	29 (19,37)	26 (20.2,35)*	23 (17.2,35)*	26 (14.3,38.6)	22 (15.8,30.2)†	28 (24,48)	23 (18,39)	18 (15,50)
LF (ms^2^)	225 (155,335)	205 (148,305)*	179 (153,295)*	252 (139,376)	162 (131,231)†	217 (177,279)	191 (152,279)*	146 (137,228)*
HF (ms^2^)	90 (69,128)	85.6 (73.3,136)*	82.5 (73,169)	87.1 (62,187)	80.6 (60.3,106)*	80 (74,192)	99 (74,120)	71 (60,107)
LF/HF	2.2 (1.7,2.9)	2.3 (1.6,2.9)*	2.3 (1.9,2.5)*	2.2 (1.7,2.85)	2.0 (1.78,2.36)*	1.9 (1.7,2.8)	2.2 (1.7,2.5)*	2.3 (2.1,2.7)
E1	1.77 (1.39,2.24)	1.74 (1.37,2.09)*	1.76 (1.35,2.18)	1.47 (1.01,1.98)*	1.62 (1.26,1.98)*	1.78 (1.6,2.36)	1.84 (1.57,2.06)	1.54 (1.14,1.9)
E2	2.16 (1.72,2.47)	2.08 (1.61,2.4)†	1.9 (1.65,2.46)	1.69 (1.2,2.27)*	1.9 (1.5,2.29)*	2.07 (1.81,2.6)	2.14 (1.87,2.34)	1.66 (1.44,2.21)
E3	2.25 (1.8,2.61)	2 (1.56,2.28)†	2.03 (1.59,2.55)*	1.69 (1.27,2.29)*	1.88 (1.52,2.23)†	2.16 (2.01,2.72)	2.15 (1.89,2.42)	1.63 (1.47,2.3)
E4	2.26 (1.75,2.62)	1.96 (1.7,2.42)†	2.05 (1.56,2.53)*	1.78 (1.36,2.41)*	1.81 (1.57,2.16)†	2.18 (2.09,2.82)	2.1 (1.95,2.5)	1.8 (1.53,2.29)
E5	2.39 (1.95,2.72)	2.18 (1.91,2.61)*	2.17 (1.69,2.75) *	1.9 (1.38,2.53)*	1.99 (1.65,2.4)†	2.27 (2.18,2.78)	2.24 (2.02,2.53)	1.93 (1.67,2.38)
E6	2.45 (2.17,2.86)	2.31 (1.98,2.7)†	2.33 (1.82,2.75) *	1.96 (1.43,2.56)*	2.09 (1.69,2.45)†	2.39 (2.27,2.76)	2.34 (2.15,2.64)	2.08 (1.68,2.49)
E7	2.47 (2.15,2.88)	2.22 (2.11,2.72)†	2.35 (1.86,2.63)*	2.02 (1.52,2.61)*	2.16 (1.77,2.45)†	2.45 (2.37,2.91)	2.36 (2.16,2.69)	2.1 (1.66,2.57)
E8	2.51 (2.12,2.87)	2.2 (2.05,2.68)†	2.35 (1.9,2.76)*	2.07 (1.59,2.62)*	2.18 (1.84,2.43)†	2.5 (2.38,2.95)	2.37 (2.12,2.72)	2.08 (1.78,2.56)

HRV Feature abbreviations: mRR: mean RR, SDNN: Standard deviation of normal RR, SD1, SD2: short and long term correlations respectively, standard descriptors of the Poincaré plot, LF: low frequency power, HF: high frequency power, LF/HF: Ratio of low frequency power over high frequency power, E1-8: Multi-Lag Entropy (lag 1 to 8). Values are presented in median (1^st^ Q, 3^rd^ Q). Numbers in brackets represent number of patients in each group.

^†^: significantly different from CONT (p< 0.01)

*: significantly different from CONT (p<0.05)

Groups that included retinopathy patients showed a significant difference in LF and LF/HF as compared to CONT. Overall, spectral indices were lower than those found for the ALL-C group. Nephropathy as a single complication had a significantly lower LF, while no HRV feature differed significantly between DPN and the control group. LF was lower for all groups investigated as compared to the control and was significant for five of the seven groups investigated.

**Fig 2:**
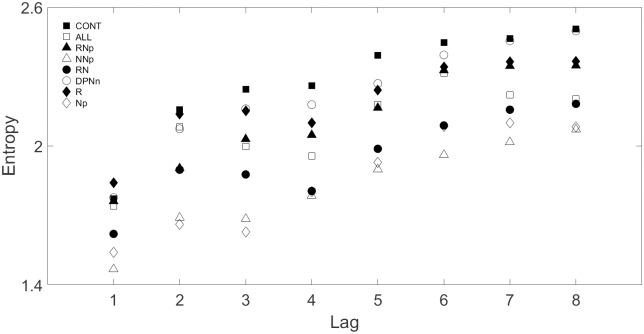
Multi-lag entropy of the 8 T2DM groups for lags 1–8.

For the multi-lag entropy analysis, significant differences were only obtained when the groups with multiple diabetic complications (ALL-C, RNp, NNp, and RN) were compared to the CONT group. However, the RNp group showed significant difference from the CONT at lags higher than 2. Fig 2 shows the multi-lag entropy for the 8 T2DM groups.

**Fig 3:**
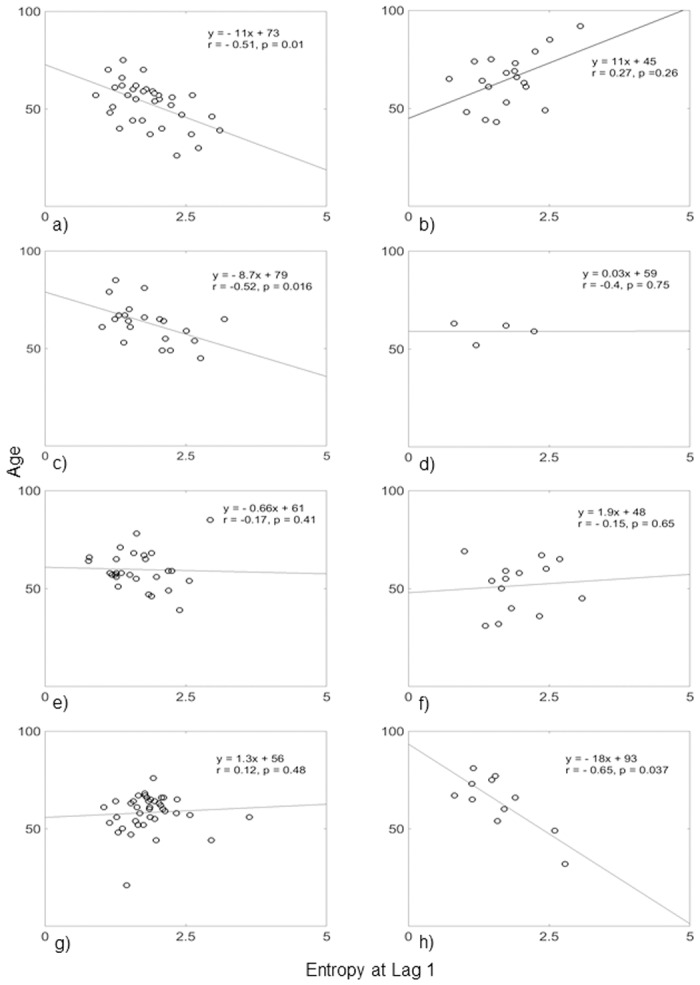
Correlation between age and entropy at lag 1 for the 8 groups. a) CONT, b) ALL-C, c) RNp, d) NNp, e) RN, f) DPNn, g) R, n) Np. Only CONT, RN and Np groups showed significant correlation (p<0.05).

In addition, we investigated whether any of the HRV measures were correlated with demographic variables using Spearman’s Rank-Order correlation (Tables 3, 4 and 5). Our results indicate that HRV features were negatively correlated with age apart from the ALL-C HRV features, which were positively correlated with age (Table 3). The highest correlations were found for the Np group (-0.65 to -0.85, p<0.05) with entropy lags above 2. Significant correlations between age and the multi-lag entropy (at all lag) were also found for the CONT, and RNp groups. An example for the Spearman’s Rank-Order correlation for multi-lag entropy (lag 1) with age for the eight patient groups is shown in Fig 3. A significant correlation with age was observed for patients with all three complications present (Fig 3a), retinopathy and nephropathy (Fig 3c) and nephropathy (Fig 3h).

**Table 3:** HRV feature correlations with age for the 8 T2DM groups.

HRV Features	CONT	ALL-C	RNp	NNp	RN	DPNn	R	Np
mRR	0.26	0.37	0.21	0.2	-0.38	0.13	0.34*	-0.4
SDNN	-0.26	0.12	-0.32	0.2	-0.39*	-0.15	-0.09	-0.65*
SD1	-0.5*	0.19	-0.52*	-0.4	-0.19	-0.21	0.11	-0.65*
SD2	-0.22	0.04	-0.29	0.2	-0.41*	-0.12	-0.1	-0.6
LF	-0.23	0.07	-0.23	0.2	-0.41*	-0.28	0.09	-0.8†
HF	-0.31	0.19	-0.52*	0.2	-0.28	-0.12	0.12	-0.48
LF/HF	0.19	-0.09	0.29	0.6	-0.32	-0.45	0.1	-0.69*
E1	-0.51*	0.28	-0.52*	-0.4	-0.17	-0.15	0.12	-0.65*
E2	-0.51*	0.16	-0.59†	-0.4	-0.18	-0.3	-0.01	-0.71*
E3	-0.54†	0.05	-0.63†	-0.4	-0.24	-0.35	-0.16	-0.84†
E4	-0.5*	0.004	-0.49*	-0.4	-0.28	-0.57	-0.14	-0.85†
E5	-0.47*	0.07	-0.53*	0.2	-0.24	-0.38	-0.14	-0.83†
E6	-0.55†	-0.01	-0.55†	0.2	-0.23	-0.36	-0.14	-0.85†
E7	-0.49*	0.04	-0.47*	0.2	-0.25	-0.39	-0.15	-0.85†
E8	-0.5*	0.02	-0.46*	0.2	-0.31	-0.35	-0.12	-0.84†

^†^: significantly different from CONT (p< 0.01)

*: significantly different from CONT (p<0.05)

**Table 4:** HRV feature correlations with DM years for the 8 T2DM groups.

HRV Features	CONT	ALL-C	RNp	NNp	RN	DPNn	R	Np
mRR	0.07	0.41	0.45*	0.2	-0.08	0.04	0.15	0.1
SDNN	-0.32	-0.19	0.18	0.2	-0.01	-0.27	-0.11	-0.04
SD1	-0.25	0.19	0.1	-0.4	-0.2	-0.16	-0.33	0.17
SD2	-0.32	-0.29	0.18	0.2	-0.003	-0.25	-0.1	-0.08
LF	-0.15	-0.06	0.3	0.2	-0.02	-0.28	-0.15	0.22
HF	-0.21	0.39	0.23	0.2	-0.12	-0.1	-0.29	-0.03
LF/HF	-0.01	-0.39	-0.26	0.6	-0.06	-0.39	0.11	-0.02
E1	-0.29	0.27	0.13	-0.4	-0.15	-0.13	0.3	0.09
E2	-0.24	0.17	0.06	-0.4	-0.18	-0.28	-0.41*	0.15
E3	-0.23	-0.09	-0.06	-0.4	-0.12	-0.34	-0.42*	0.17
E4	-0.24	-0.06	0.01	-0.4	-0.23	-0.62*	-0.24	0.13
E5	-0.21	-0.06	0.03	0.2	-0.12	-0.41	-0.35*	0.11
E6	-0.23	-0.1	-0.09	0.2	-0.06	-0.38	-0.43*	0.13
E7	-0.22	-0.16	-0.03	0.2	-0.14	-0.45	-0.39*	0.07
E8	-0.24	-0.1	-0.05	0.2	-0.12	-0.41	-0.29	0.12

*: significantly different from CONT (p<0.05).

**Table 5:** HRV feature correlations with systolic blood pressure, high density lipoprotein level and total cholesterol level for CONT group.

HRV Features	SBP	HDL	TC
mRR	-0.34	-0.1	-0.2
SDNN	-0.32	-0.57†	-0.39
SD1	-0.5*	-0.29	-0.15
SD2	-0.31	-0.56†	-0.43*
LF	-0.41*	-0.59†	-0.45*
HF	-0.46*	-0.21	-0.08
LF/HF	0.12	-0.34	-0.46*
E1	-0.46*	-0.23	-0.14
E2	-0.45*	-0.3	-0.09
E3	-0.44*	-0.42*	-0.16
E4	-0.46*	-0.54†	-0.34
E5	-0.42*	-0.51*	-0.38
E6	-0.43*	-0.52†	-0.29
E7	-0.42*	-0.51*	-0.25
E8	-0.4	-0.51*	-0.29

SBP: Systolic Blood Pressure, HDL: High Density Lipoprotein, TC: Total Cholesterol.

^†^: indicate significant correlation (p< 0.01)

*: indicate significant correlation (p<0.05)

Years of diabetes did not significantly correlate with all HRV features (Table 4). Only the entropy features from lag 2 to lag 7 and presence of retinopathy and DPN had any degree of correlation with years of diabetes. The highest significant correlation was found between the presence of DPN and entropy at lag 4 (-0.62) followed by a correlation of -0.43 for retinopathy and entropy at lag 6. Some of the clinical variables and HRV features were also significantly correlated but only in the CONT group (Table 5).

High density lipoprotein (HDL) showed the highest correlation with LF power. All correlations were negative and in agreement with clinical observations, where abnormally high systolic blood pressure or cholesterol levels affects heart rhythm negatively.

The OR in Table 6 indicate that the time and frequency features had an OR of 1, thereby suggesting no association or increased risk of diabetes complications, except for the LF/HF feature, which had OR values between 1.33 and 4.92. The highest OR was found for the ALL-C group. In contrast the OR for the multi-lag entropy had values less than 1 except for RN and entropy lags between 4 and 8. OR between 0.4 and 0.7 were obtained for the R group and between 0.2 and 0.4 for the DPNn group. The combination of retinopathy and nephropathy (RNp group) lowered the OR (0.15–0.4), whereas the combination of all three complications again lowered the OR (0.5–0.7). The OR between the RN group and the other complication groups (Table 7) shows that the differences in ANS dysfunction between the retinopathy-DPN and retinopathy-nephropathy complications are manifested more on the multi-lag entropy measure than the LF/HF ratio. To study the influence of gender on the results we stratified data from the CONT and the RN group (the group that showed most significant difference from CONT) by sex. The male groups (CONT: 12, RN: 6 subjects) had matched age and DM years while the female CONT group had significantly lower DM years, p<0.01 (CONT: 22, RN: 21 subjects). However, no significant differences were found in any of the HRV features between the male and female groups.

**Table 6:** Odds ratio of the HRV features between the CONT and DM with complications groups.

Feature	ALL-C	RNp	RN	DPNn	R
mRR	1	1	1	1	1
SDNN	0.92	0.98	1	0.95	0.99
SD1	0.97	0.89	1	0.95	0.97
SD2	0.94	0.99	1	0.96	1
LF	1	1	1	1	1
HF	1	1	1	1	1
LF/HF	4.92	1.35	1.78	1.36	1.33
E-1	0.73	0.17	0.91	0.39	0.6
E-2	0.67	0.18	0.89	0.35	0.44
E-3	0.62	0.14	0.86	0.23	0.39
E-4	0.57	0.3	1.17	0.24	0.56
E-5	0.55	0.26	1.42	0.31	0.55
E-6	0.53	0.23	1.38	0.27	0.51
E-7	0.51	0.39	1.18	0.39	0.62
E-8	0.68	0.39	1.42	0.22	0.7

**Table 7:** Odds ratio of the HRV features between the RN group and the other DM with complications groups.

Feature	ALL-C	RNp	DPNn	R
mRR	1	1	1	1
SDNN	1.05	1.03	0.94	0.97
SD1	1.01	1.07	0.85	0.95
SD2	1.03	1.02	0.96	0.98
LF	1	1	1	1
HF	1	1	0.99	1
LF/HF	2.08	1.43	2.12	0.83
E-1	1	2.2	0.2	0.5
E-2	1.07	1.99	0.18	0.41
E-3	0.86	2.05	0.15	0.43
E-4	1.17	2.31	0.11	0.33
E-5	1.29	2.62	0.18	0.27
E-6	1.23	2.69	0.14	0.33
E-7	1.18	1.95	0.1	0.39
E-8	1.25	2.46	0.09	0.37

## Discussion

Clinical studies have shown that development of T2DM complications is definitely linked to hyperglycemia, but the pathophysiology of retinopathy, nephropathy and DPN are different [4, 33]. DPN which occurs in up to 60% of long-term T2DM, can lead to cardiac arrhythmias and sudden cardiac death [34]. Similarly diabetic retinopathy and nephropathy have been linked with cardiovascular events [35, 36]. Our current study investigated the relationship between DPN, diabetic retinopathy and nephropathy specific T2DM complications and combinations of these complications with the cardiac autonomic nervous system by using traditional and multi-lag HRV analysis in a previously not studied Arab Emirati T2DM cohort. The prevalence of DPN in this study was 38%, which is comparable with data from other T2DM populations from Saudi Arabia (38%) [37], Mexico (40%) [38], UK (36%) [39] and Egypt (22%) [40]. Nephropathy population in this study (33%) was also comparable to other studies from Jordan (33%) [41], Egypt (42%) [40] and Saudi Arabia (40%) [42]. Retinopathy prevalence (63%) was higher than reported results from Saudi Arabia (31%) [43], Oman (14.4%) [44] and Yemen (55%) [40].

The pathophysiology of diabetic complications also includes dysfunction of the ANS, which also affects cardiac function and is the main cause of cardiovascular morbidity and mortality in T2DM [34]. Diabetic associated complications are typically considered to be a function of disease duration, which is reflected in ANS dysfunction, where the parasympathetic division is usually affected earlier compared to sympathetic dysfunction. However the role of age, years of diabetes and the interaction between different types of diabetic complications with cardiac autonomic dysfunction has not been investigated previously. Traditional clinical features including age and the number of years of T2DM differed significantly between the diabetic complication groups suggesting a multifactorial model for progression in diabetes related complications. Lifestyle factors, such as poor glycemic control, diet, obesity and hypertension have been shown to lead to earlier onset of diabetic complications [45]. Our results confirm these findings and further bring attention to the differences in HRV and some anthropometric and clinical data parameters with respect to different diabetic complications. The DPNn group was the youngest and the nephropathy group the oldest in our cohort. The older age of the nephropathy group may reflect age related changes in addition to diabetes, which play a role in development of nephropathy, especially as the mean years of diabetes in this group was the lowest and similar to the control group with no complications. These age-related changes could explain the significantly lower LF power in the CONT as compared to the other groups having only one complication. Blood pressure, BMI and HbA1c were all above normal in the nephropathy group, which also play an important role in the pathogenesis of diabetic nephropathy. Patients with all three complications had on average over 15 years of disease duration. Our findings show that retinopathy is more likely to occur as a single lesion later during disease progression, which differs to previous studies. In the Early Treatment Diabetic retinopathy Study approximately 20% of T2DM patients already had retinopathy at the onset of diabetes [46]. When combined with DPN, the average age of the cohort was younger bringing the age closer to the DPN cohort (Table 1). This suggests a strong link between DPN and retinopathy, with possibly large vessels being more susceptible to the effects of hyperglycemia followed by small vessel disease such as retinopathy and diabetic nephropathy in the current cohort. Single diabetes complications were significantly correlated with age (nephropathy) and DM years (retinopathy) in the current study. This agrees with previous clinical research on T1DM [47]. However associations between retinopathy and nephropathy in T2DM have not been fully explored [48]. Combinations of diabetic complications resulted in a different set of significant correlations with age. Of these only the ALL-C group was positively correlated with age. The positive correlation with age and the most severe affected diabetes complication groups explains why higher HRV indices do not always reflect better cardiac autonomic function and are similarly to lower HRV values signs of autonomic pathology [49]. All groups had increased HbA1c above recommended levels, indicating that glucose control is an important factor in developing diabetic complications [50]. Significant correlations between HRV features and clinical variables as found in this study and previously in a Caucasian cohort suggest that HDL cholesterol may also be contributing to HRV, followed by systolic blood pressure [51, 52].

The mean age of the DPNn group was the lowest despite a relative long T2DM duration similar to the remaining diabetic complications groups. Of interest is that this group had the highest mean HbA1c suggesting that peripheral nerve function is more susceptible to uncontrolled diabetes but may be also exacerbated by the type of footwear most often worn in the Middle East, where wearing open toes sandals can lead to foot deformity and loss of microvascular flow to the foot [53]. DPN is also associated with autonomic nervous system dysfunction. Symptoms include postural hypotension, abnormal sweating, gastrointestinal and urinary dysfunction, which often is not noticed by the patients [54]. Including tests for autonomic neuropathy such as HRV analysis of the cardiac rhythm are therefore an important option for diagnosis of diabetic complications and identification of ANS associated pathology [55, 56].

With respect to the type of HRV analysis most useful in clinical practice, we have recently reported that multi-lag entropy analysis addresses limitations of single lag (beat-to-beat) entropy analysis in HRV studies [57]. Multi-lag analysis considers that a heartbeat influences not only the beat immediately following it and identified by single-lag analysis, but also up to 6–10 beats downstream, which requires multi-lag analysis [58]. Our HRV results show that impairment of the ANS, characterized by decreasing time and frequency domain HRV results as well as the entropy HRV features, clearly increases as patients have more than one complication and provides a better understanding of whether the changed heart rate variability is primarily based on vagal (lags 1–6) or a combination of sympathetic and parasympathetic dysfunction (lags 7–10).

The majority of the correlation results of diabetic complications with years of DM were negative suggesting that with increasing years of diabetes, the risk of ANS dysfunction increases, but was only significant in the retinopathy group. In terms of single complications, it is interesting to observe that DPN patients showed the least difference in HRV compared to the CONT group, despite recent results that DPN is strongly associated with ANS dysfunction and suggests large fiber involvement with vascular pathology including cardiovascular disease (CVD) [54]. This lack of association found in the current study may be due to small fibre disease having a different aetiology to large fibre disease. Retinopathy patients had significantly lower LF, and LF/HF results compared to the CONT group indicating a possible sympathovagal dysfunction. The effect of the increase in the number of diabetic complications was also manifested in the multi-lag entropy, which decreased (except for the case of nephropathy). The entropy evaluates total acceleration-inhibition activities of the heart rate, or total heart period variation. The multi-lag entropy analysis gives additional information on the short-term (beat-to-beat) and long-term variations in HRV associated with parasympathetic and sympathetic function related to diabetes complications.

The relationship between autonomic impairment and diabetic complications was also seen when odds ratios were determined. The OR for all complications being present determined and LF/HF was nearly 4 times greater compared to the OR for presence of any single or multiple complications. When retinopathy was combined with DPN, the OR at higher lags of the entropy measure was significant suggesting that long-term changes in heart rate was more associated with sympathetic dysfunction. The accurate detection of sympathetic dysfunction is crucial but conventional HRV features are not sensitive to the sympathetic component [11]. Multi-lag entropy however is able to resolve sympathetic function by including higher lags in the analysis that are unlikely to contain a parasympathetic component. This was supported by the higher OR in the multi-lag measure (compared to the conventional LF/HF ratio) between the RN and the RNp groups.

## Conclusion

The results of this study offer a novel approach to detecting both parasympathetic and sympathetic changes that characterize differences in patients with single or multiple diabetes complications. Our findings further characterize a local group of UAE citizens with diabetes attending a hospital diabetes outpatient clinic and hence provide a database to evaluate the influence of diabetic complications in this ethnic group.

## Abbreviations

ANS: Autonomic nervous system
ALL-C: T2DM patients with retinopathy, nephropathy and DPN
BGL: Blood Glucose Level
BMI: Body Mass Index
CAN: Cardiac autonomic neuropathy
CONT: T2DM patients with no retinopathy, nephropathy or DPN
DBP: Diastolic Blood Pressure
DPN: Diabetic peripheral neuropathy
DPNn: T2DM patients with DPN
HbA1c: Glycated Hemoglobin Level
HDL: High Density Lipoprotein
HF: High frequency power of HRV
HRV: Heart rate variability
LDL: Low Density Lipoprotein
LF: Low frequency power of HRV
mRR: Mean of normal RR intervals
NNp: T2DM patients with DPN and nephropathy
Np: T2DM patients with nephropathy
OR: Odds ratio
PI: Percentage index of changes in successive RR intervals
R: T2DM patient with retinopathy
RN: T2DM patients with retinopathy and DPN
RNp: T2DM patients with retinopathy and nephropathy
SBP: Systolic Blood Pressure
SD1, SD2: Standard descriptors of the Poincaré plot
SDNN: Standard deviation of normal RR intervals
T2DM: Type 2 Diabetes Mellitus
TC: Total Cholesterol
